# The role of microRNAs in pathophysiology and diagnostics of metabolic complications in obstructive sleep apnea patients

**DOI:** 10.3389/fnmol.2023.1208886

**Published:** 2023-07-21

**Authors:** Filip Franciszek Karuga, Julia Jaromirska, Mikołaj Malicki, Marcin Sochal, Bartosz Szmyd, Piotr Białasiewicz, Dominik Strzelecki, Agata Gabryelska

**Affiliations:** ^1^Department of Sleep Medicine and Metabolic Disorders, Medical University of Lodz, Lodz, Poland; ^2^Department of Neurosurgery and Neuro-Oncology, Barlicki University Hospital, Medical University of Lodz, Lodz, Poland; ^3^Department of Pediatrics, Oncology, and Hematology, Medical University of Lodz, Lodz, Poland; ^4^Department of Affective and Psychotic Disorders, Medical University of Lodz, Lodz, Poland

**Keywords:** microRNA, OSA, obstructive sleep apnea, diabetes, metabolic syndrome, metabolic complications

## Abstract

Obstructive sleep apnea (OSA) is one of the most common sleep disorders, which is characterized by recurrent apneas and/or hypopneas occurring during sleep due to upper airway obstruction. Among a variety of health consequences, OSA patients are particularly susceptible to developing metabolic complications, such as metabolic syndrome and diabetes mellitus type 2. MicroRNAs (miRNAs) as epigenetic modulators are promising particles in both understanding the pathophysiology of OSA and the prediction of OSA complications. This review describes the role of miRNAs in the development of OSA-associated metabolic complications. Moreover, it summarizes the usefulness of miRNAs as biomarkers in predicting the aforementioned OSA complications.

## 1. Introduction

Obstructive sleep apnea (OSA) is a chronic breathing disorder, which presents with recurrent apneas and/or hypopneas during sleep (Arnold et al., 2017). As the result of neuromuscular factors (e.g., hypoglossal nerve and genioglossus muscle) and negative airway pressure the tongue falls backward leading to occlusion in the upper airway (Pham and Schwartz, 2015). Apnea is a cessation of breathing for at least 10 s, while hypopnea means a reduction in the airflow by at least 50% associated with a drop in arterial blood oxygen saturation of at least 3% or reduction of the airflow by 30% and desaturation by 4% (Krawczyk et al., 2013). The gold standard in diagnosing and assessing the severity of OSA is the nocturnal polysomnography (PSG) examination. Although PSG is a prominent diagnostic tool for detecting OSA, the study can be aggravating for patients and does not inform about the risk of metabolic complications. During the PSG examination, apnea-hypopnea index (AHI) defined as the number of apneas and hypopneas per hour of effective sleep, is calculated. This index shows the severity of the disease: 5 > AHI – no OSA, 15 > AHI ≥ 5 – mild, 30 > AHI ≥ 15 – moderate, and AHI ≥ 30 – severe OSA (Kapur et al., 2017). It is estimated that 14% of men and 5% of women experience mild, moderate, or severe forms of this condition in their lifetime, however, in some populations, the percentage can reach up to 80% (Patil et al., 2019). Unfortunately, a large proportion of moderate and severe OSA, remain undiagnosed (Young et al., 1993; Fleming et al., 2018). Obesity is considered the most prominent risk factor for developing OSA, followed by male sex, and older age (Liu et al., 2016). A higher prevalence of OSA is observed in patients who have hypertension (30–83%) (Logan et al., 2001), diabetes mellitus (40–69%) (Fallahi et al., 2019), or metabolic syndrome (55–81%) (Drager et al., 2009). Dominant symptoms of OSA are non-specific: snoring, drowsiness, excessive daytime sleepiness, and fatigue. OSA is dangerous not only because of chronically occurring hypoxia but also because of its possible complications, such as diabetes mellitus type 2 (T2DM), metabolic syndrome (MetS), cardiovascular diseases, asthma, idiopathic pulmonary fibrosis, cancer (Kong et al., 2016; Cao et al., 2022; Karuga et al., 2022; Salari et al., 2022; Wang C. et al., 2022; Wang et al., 2023). Continuous positive airway pressure (CPAP), a gold standard treatment in OSA, might delay or even eliminate symptoms and complications of this disorder (Fu et al., 2017; Bonsignore et al., 2019; Gabryelska et al., 2021b). However, individuals who are diagnosed with moderate or severe OSA might require additional treatment, for example, oral appliances, hypoglossal nerve stimulators, or upper-airway surgeries (Waters, 2019). Many papers link higher mortality in OSA with metabolic complications (Labarca et al., 2021; Su et al., 2021; Liu et al., 2022). Therefore, it is crucial to detect the early signs of OSA complications in order to optimize the treatment strategy (Kendzerska et al., 2014). Ongoing research focuses on finding a satisfactory biomarker to achieve this goal. The most promising candidates for OSA biomarkers include as follows: miRNA levels of *ADAM29*, *FLRT2*, and *SLC18A3* determined in peripheral blood mononuclear cells, serum levels of Endocan and YKL-40, as well as plasma levels of IL-6 and Vimentin (Gaspar et al., 2022). Contemporary miRNA exhibits several advantages over other molecular biomarkers. It has been reported that noninvasive quantification of miRNA profiles is highly sensitive, robust, and cost-effective for the clinical management of different pathological conditions such as head and neck squamous cell carcinoma, heart failure, or osteoporosis Additionally, detecting differences in gene expression rather than in gene content becomes an effective and practical approach for associating molecular markers with the patient phenotype and disease outcome (Bock, 2009). There are miRNAs that are involved in OSA and the development of its complications (Malicki et al., 2022). Therefore, such miRNAs can reveal clinical value in order to specific OSA phenotypes diagnosis or act as a predictive factor of concrete complications development at an early stage of the disease (Gaspar et al., 2022). In the present study, we reviewed miRNAs that can be potential biomarkers of OSA metabolic consequences and should be investigated in the future. However, it should be mentioned that miRNA biomarkers have their limitations in both sensitivity and specificity. The blood sampling methods should be carefully considered as well as the selection criteria of the study group due to the influence of concomitant diseases miRNAs levels. In addition, each miRNA can have various expressions depending on the specimen (e.g., blood, urine, muscle tissue, exosomes).

MiRNAs are small, up to 30 bases in length, strands of ribonucleic acid, which are responsible for regulating the expression of many genes (Dutkowska et al., 2021). Based on the location miRNAs are generally divided into two groups – intracellular and extracellular. While intracellular mature miRNAs can be secreted from the cytoplasm and preserve high stability, extracellular molecules, e.g., packaged in exosomes or encapsulated in liposomes, are promising and more accessible for clinical diagnostics (Leung, 2015). MiRNAs can be used to detect the disease before its first manifestations and predict the response of the organism to the suggested treatment (Gabryelska et al., 2020b,c,d; Pozniak et al., 2022). The main role of miRNAs is cell-to-cell communication via post-transcriptional epigenetic mechanism leading to mRNA degradation or translation inhibition (Czarnecka et al., 2019). Their proper functioning is crucial for maintaining correct cell activity, such as apoptosis, stress response, proliferation, and metabolism. Any disruption in the regulatory properties of miRNAs can result in the pathogenesis of many disorders, including respiratory diseases and their complications (Dutkowska et al., 2021). Therefore, analyzing miRNAs, particularly as specific subsets, may be considered not only important for a better diagnosis and therapy but also as an essential factor for understanding the pathophysiology of OSA and its association with other disorders (Gabryelska et al., 2020a). In the current review, we explain the role of miRNAs in the development of OSA-related metabolic complications.

## 2. miRNAs in metabolic complications of OSA

### 2.1. OSA

A better insight into OSA-related miRNAs may lead not only to a better understanding of OSA but also to the development of new diagnostic and therapeutic strategies, as PSG examination is a good tool for diagnosing OSA but is expensive and uncomfortable for patients – they have to spend at least one night away from home. In addition, miRNA expression may affect gene expression, which can result in the development of complications, especially metabolic. Santamaria-Martos et al. (2019a) performed an analysis to determine miRNAs that separate OSA from non-OSA patients. Initially, 14 miRNAs (−*181-a-2, −495, −451, −486, −660, −345, −340, −107, −486-3p, −133a, −181a, −let-7d, −199a, −199b*) revealed different expression between these two groups. However, further validation with the qPCR method confirmed that only six of them might be suitable for clinical use (−*181a, −199b, −345, −133a, −340, −486-3p*). Khurana et al. published their cohort study where they noted the downregulation of *miRNA-27* and *let-7* in OSA patients (Khurana et al., 2020). Santamaria-Martos et al. (2019b) selected eight biomarker candidates: *miRNA-106a, miRNA-186, miRNA-29a, miRNA-21, miRNA-103, miRNA-27a, miRNA-140, and miRNA-145*. It was observed that the combination *miRNA-106a/miRNA-186* was the most stable among all the candidates (Santamaria-Martos et al., 2019b). Although some of the above-mentioned miRNAs have not been described as dysregulated particularly in diabetes mellitus or metabolic syndrome, studies are needed to specify their levels and potential role in patients with OSA and concomitant metabolic complications. Common miRNAs between OSA and metabolic complications can include also miRNAs: −17-5p, −21-5p, −22-3p, −31, −126, −130, −155, −181, and − 199; their involvement is further presented in this review.

### 2.2. Metabolic syndrome

Metabolic syndrome (MetS), initially known as syndrome X, was first described by Reaven in 1988 after it was noticed that insulin resistance and hyperinsulinemia increase the risk of T2DM, hypertension, and coronary artery disease development (Reaven, 1988). According to the most common definition, MetS is a co-occurrence of abdominal obesity and at least two cardiometabolic risk factors such as hypertension, insulin resistance, hypertriglyceridemia, and low concentration of high-density cholesterol (Saklayen, 2018). MetS increases the risk of cardiovascular complications occurrence 2-fold and up to 5-fold in the case of T2DM (Samson and Garber, 2014). The prevalence of MetS has increased worldwide with averaging values at 30% in adults (Engin, 2017) and between 6 and 39% in children/teenagers (Weihe and Weihrauch-Blüher, 2019). MetS has a variety of anticipated miRNA biomarkers, which are summarized in Table 1. Furthermore, some of the miRNAs can have a gender-specific (Wang et al., 2013) or physical (Zhou et al., 2014) link in MetS patients. Obstructive sleep apnea is independently associated with MetS occurrence. Risk estimates are 6–9 times higher in patients with OSA compared to the general (non-OSA) population (Coughlin et al., 2004). It has been shown that CPAP reduces the risk of developing MetS in OSA patients (Phillips et al., 2011). Although the connection between OSA and MetS is not fully understood, studies are emphasizing the influence of intermittent hypoxia and sleep fragmentation (Khalyfa et al., 2018), abnormal sympathetic activation (Trombetta et al., 2013), and chronic inflammation (Drager et al., 2010; Turkiewicz et al., 2021; Kaczmarski et al., 2022). There are no conducted studies focusing on the relationship between patients with MetS and OSA in the context of miRNAs. Nevertheless, preliminary conclusions can be drawn from the available literature.

**Table 1:** The role of selected miRNAs in metabolic syndrome and obstructive sleep apnea pathogenesis.

MicroRNA	Up/Downregulated in OSA and MetS	Predicted target	Implications	Reference
*miR-17-5p*	⬇	*TXNIP*	⬆β-cell death	Koenen et al. (2011), Masutani et al. (2012), and Ramzan et al. (2020)
*miR-21-5p*	⬇	*TLR4*	⬆Low-grade inflammation	Boutagy et al. (2016), Sapp et al. (2019), and Ali et al. (2021)
*miR-22-3p*	⬇	*GR* *IL6R*	⬆Cortisol levels ⬆IL-6 levels	Huang et al. (2020)
*miR-130*	⬆	*PPAR-γ*	⬆Production of reactive oxygen species	Kim et al. (2013) and Rega-Kaun et al. (2020)
*miR-181*	⬇	*TLR4*	⬆Low-grade inflammation	Hulsmans et al. (2012)

GR, glucocorticoid receptor; IL6R, interleukin 6 receptor; PPAR-γ, peroxisome proliferator-activated preceptor gamma; TLR4, toll-like receptor 4; TXNIP, thioredoxin-interacting protein; OSA, obstructive sleep apnea.

The dysregulation of *miRNA-181a* presented in OSA may exacerbate inflammation in the case of MetS as a comorbid condition (Santamaria-Martos et al., 2019b). Hulsmans et al. study identified toll-like receptor (TLR) 4 as an inflammation factor associated with the levels of *miRNA-181a* in patients with both morbid obesity and MetS; the downregulation contained in CD14+ monocytes enhanced TLR/NF-κB signaling pathway, which was associated with chronic low-grade inflammation development (Hulsmans et al., 2012). The target gene may be a TLR4-interactor with leucine-rich repeats, a functional component of TLR4. The knockdown of TLR4 via siTLR4 has been shown to affect the *miRNA-181a* levels (Xie et al., 2013b). As the decreased levels of *miRNA-181a* in monocytes are associated with targeting IL-1 via binding the site of 3′-untranslated regions, the modulation of miRNA can alleviate systemic inflammation and thereupon decrease the severity of both MetS and OSA or even act as MetS development prevention (Xie et al., 2013a).

Altered *miRNA-22-3p* expression in OSA patients (Shao et al., 2021) can contribute to greater severity of MetS since it takes part in the development of the main features of the disease. *MiRNA-22-3p* has been reported to be downregulated in peripheral blood mononuclear cells (PBMCs) in MetS patients – the study disclosed a negative correlation with a variety of MetS components such as blood pressure, plasma triglyceride, and waist circumference, and a positive correlation with plasma high-density lipoprotein levels (Huang et al., 2020). Furthermore, OSA-related hypertension may arise from *miRNA-22-3p* downregulation that influences vascular smooth muscle cells (VSMCs) by targeting the methyl-CpG binding protein 2 gene (Shao et al., 2021). Excessive proliferation and migration of VSMCs impede the arterial intima balance, which is inherently connected with atherosclerosis and subsequent hypertension development, thus pointing at *miRNA-22-3p* dysregulation as a trigger for the development of hypertension.

The downregulation of *miRNA-17-5p* might be a novel biomarker of OSA or MetS, which is linked with obesity and impaired adipogenesis (Ramzan et al., 2020). Thioredoxin interacting protein (TXNIP) is an aspiring target of *miRNA-17-5p* which was described in metabolic disorders including obesity and insulin resistance (Koenen et al., 2011; Masutani et al., 2012). Exposing the genioglossus muscle cells to intermittent hypoxia led to a downregulation of *miRNA-17-5p*. Upregulation of *miRNA-17-5p* by miRNA mimics resulted in a strengthening of the muscle by regaining the mitochondrial function and cell proliferative capacity (Qin et al., 2019). Genioglossus plays an important role as an upper-airway dilator muscle, helping to maintain adequate oxygen supply during sleep. TXNIP may have an impact on tissue subjected to chronic intermittent hypoxia via TXNIP/NLRP3/IL-1β pathway and mediate mitochondrial dysfunction (Yan et al., 2021). Thus, the bidirectional relationship can be observed – downregulation of *miRNA-17-5p* occurring during OSA may exacerbate the likelihood of MetS occurrence through affecting fat accumulation, while downregulation happening in MetS may weaken the restoration power of genioglossus muscle and make the patient more susceptible to apnea development or cause a more severe course of coexisting OSA.

As mentioned before, some miRNAs might be used as potential diagnostic markers only in a specific group. Sapp et al. study revealed downregulation of *miRNA-21-5p* in postmenopausal African American women with MetS compared to a healthy group (Sapp et al., 2019). These results contrast with those found in a study by Doghish et al. where *miRNA-21-5p* levels were increased in adult Egyptian males with MetS (Doghish et al., 2021). In treatment-naive OSA patients, *miRNA-21-5p* was decreased and negatively correlated with oxygen desaturation index and AHI. Exposure of human monocytic THP-1 cell lines (cell model of human monocytes) to intermittent hypoxia with reoxygenation resulted in decreased levels of *miRNA-21-5p*, *in vitro* studies pointed out that *miRNA-21-5p* might target TNF-α/TLR4 pathway resulting in hypoxia-induced inflammation and cell apoptosis (Chen Y. C. et al., 2020). In turn, TLR4 activation in MetS may increase reactive oxygen species production, which has been related to inflammation, endothelial dysfunction, and metabolic impairment (Boutagy et al., 2016; Ali et al., 2021). The downregulation of *miRNA-21-5p* in OSA and MetS may indicate a self-perpetuating cycle of inflammation via activation of TLR4 as a pattern recognition receptor, and cell dysfunction. However, this was observed only in specific groups, prompting the need for further investigation.

Altered expression of *miRNA-130* might be relevant to MetS and indicate metabolic aspects of OSA. In tissue exposed to chronic intermittent hypoxia, the major harmful factor of OSA, the *miRNA-130* levels were upregulated (Zhang X. B. et al., 2020). The study of Rega-Kaun et al. (2020) showed that Roux-en-Y-Bariatric Surgery significantly reduced upregulated *miRNA-130* levels in MetS patients, which exhibited clinical improvement of the disorder. Elevated *miRNA-130* can especially affect islets of Langerhans via modulation of ATP/ADP concentration, thereby impairing the leading pancreatic function relying on glucose homeostasis regulation (Ofori et al., 2017). Additionally, in obese schoolchildren aged 12–18 *miRNA-130* positively correlated with MetS risk factors (Al-Rawaf, 2019). Peroxisome proliferator-activated receptor gamma (PPAR-γ) was found to be a possible target of *miRNA-130* – PPAR-γ might regulate adipocyte differentiation and promote both oxidative stress injury and proinflammatory response (Kim et al., 2013; Zhang Y. et al., 2017). Especially in a chronic intermittent hypoxic environment, PPAR-γ is in charge of neuroinflammation development and cognitive performance (Dong et al., 2018; Wang H. et al., 2021), endothelial cell regulation (Lian et al., 2021), and defense from the kidney (Zhang et al., 2019) or cardiac (Pai et al., 2022) injury. Nocturnal hypercapnia, another hallmark of OSA, can contribute to obesity and metabolic impairment through PPAR-γ dysregulation (Kikuchi et al., 2017). Overall, *miRNA-130* can be a valuable predictor in children/adolescents of subsequent MetS and OSA development and a biomarker of treatment response in adults.

**Figure 1:**
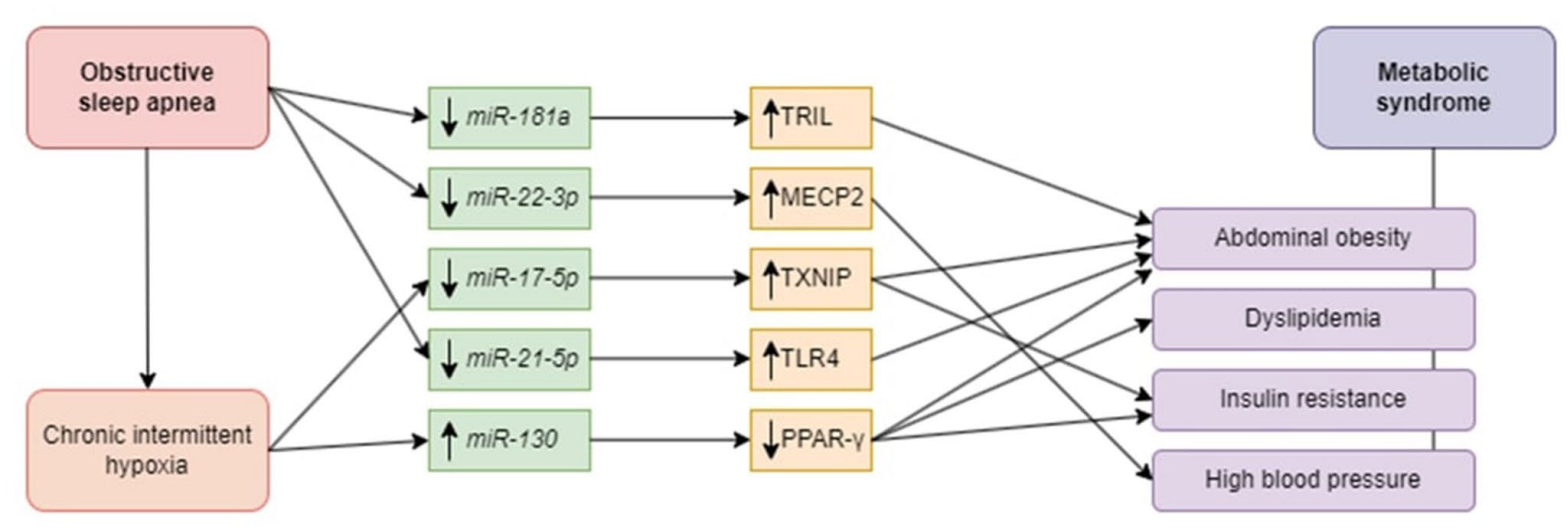
Potential role of microRNAs in the pathophysiology of metabolic syndrome in obstructive sleep apnea patients. Obstructive sleep apnea in general affects *miR -181a, -22-3p, -17-5p*, and *via* chronic intermittent hypoxia *miR-21-5p* and -*130*. Downregulation of *miR-181a* causes upregulation of TRIL and leads to abdominal obesity. Downregulation of *miRNA-22-3p* is associated with high blood pressure due to targeting MECP2. Downregulation of *miR-17-5p* triggers abdominal obesity and insulin resistance *via* TXNIP upregulation. *miR-21-5p* downregulation leads to the upregulation of TLR4, thereby contributing to abdominal obesity. Upregulation of *miRNA-130* stimulates PPAR-γ, predisposing to abdominal obesity, dyslipidemia, and insulin resistance. miR, microRNA; TRIL, TLR4 interactor with leucine-rich repeats; MECP2, methyl-CpG binding protein 2; TXNIP, thioredoxin interacting protein; TLR4, toll-like receptor 4; PPAR- γ, peroxisome proliferator-activated receptor gamma.

The role of chosen miRNAs in the development of OSA-related metabolic complications is presented in Figure 1.

### 2.3. Diabetes mellitus

Diabetes mellitus is a group of metabolic disorders characterized by chronic hyperglycemia resulting from defects in insulin action, insulin secretion, or both. T2DM is the most common type of diabetes mellitus. In 2017, approximately 6.28% of the world’s population was affected by T2DM and around 1 million deaths yearly can be attributed to T2DM alone(Khan et al., 2020). The risk factors for T2DM include both nonmodifiable factors (e.g., genetic predisposition, family history), and modifiable factors (e.g., obesity, unhealthy diet, and low physical activity) (Galicia-Garcia et al., 2020). There are many miRNAs that may have an impact on T2DM (see Table 2). They can inhibit insulin signaling, inhibit glucose uptake, promote insulin signaling, and reduce insulin secretion(Sekar et al., 2016; Szweda and Łaczmański, 2016; Rovira-Llopis et al., 2018; Deng and Guo, 2019; Kim and Zhang, 2019; Jankauskas et al., 2021). OSA is recognized as an independent risk factor for metabolic diseases, including T2DM (Gabryelska et al., 2020a). In the study of Mahmood et al., it was found that the prevalence of T2DM in OSA patients was 30.1%, while in the group without OSA only 18.6% (Mahmood et al., 2009). Moreover, Gabryelska et al. (2021a) showed that higher oxygen saturation in OSA patients is associated with the later onset of T2DM. As it was mentioned before the most effective form of treatment for OSA is CPAP, which generates air pressure in the upper airways preventing their collapse and eliminating the recurrent periods of hypoxia. Unfortunately, CPAP treatment might be ineffective in patients with T2DM (Labarca et al., 2018), however, it may slow down the progression of T2DM (Reutrakul and Mokhlesi, 2020). The coexistence of these two diseases is the subject of numerous scientific papers, which investigate possible mechanisms of this interaction, for example, mechanisms mediated via HIF-1α or sirtuin1 (SIRT1) (Reutrakul and Mokhlesi, 2017; Song et al., 2018; Gabryelska et al., 2020a). Nevertheless, the relationship between these two diseases is not fully understood. Some studies postulate the role of certain miRNAs in T2DM among OSA patients.

**Table 2:** The role of selected miRNAs in diabetes mellitus and obstructive sleep apnea pathogenesis.

MicroRNA	Up/Downregulated in OSA and T2DM	Predicted target:	Implications	Reference
*miR-31*	⬆	*SATB2*	Endothelial dysfunction	Lian et al. (2018)
			Impaired bone remodeling	Zhen et al. (2017)
*miR-126*	⬇	*SPRED1*	Endothelial dysfunction	Meng et al. (2012) and Rezk et al. (2016)
		*CASP3*	Diabetic retinopathy	Chen X. et al. (2020)
		*VEGF*		Ye et al. (2013)
*miR-155*	⬆	*FOXO1*	Diabetic nephropathy	Wang G. et al. (2020)
		*PTEN*		Guo et al. (2020)
		*BDNF*		Gao et al. (2022)
		*SIRT1*		Wang et al. (2018) and Wang X. et al. (2021)
		*TP53*	Diabetic cardiomyopathy	Raut et al. (2016)
		*PDCD4*		Zhao S. F. et al. (2021)
		*KLF6*	Diabetic nephropathy	Liang and Xu (2020)
		*EGR1*		Xu et al. (2017) and Zha et al. (2019)
*miR-181a*	⬇	*PDCD4 TP53*	Diabetic cardiomyopathy	Raut et al. (2016) and Zhao S. F. et al. (2021)
		*EGR1 KLF6*	Diabetic nephropathy	Zha et al. (2019b) and Liang and Xu (2020)
*miR-199a*	⬇	*SP1*	Diabetic cataract	Liu et al. (2020)
		*VEGF*	Diabetic retinopathy	Wang L. et al. (2020)
		*FGF7*		Zhou et al. (2021)
		*IKKβ*	Diabetic nephropathy	Zhang R. et al. (2020)
		*AKT1, AKT2, VEGF, IGF1, FGF1*	Diabetic cardiomyopathy	Ahmed et al. (2021)

AKT1, AKT serine/threonine kinase 1; AKT2, AKT serine/threonine kinase 2; BDNF, brain derived neurotrophic factor; CASP3, caspase 3; EGR1, early growth response 1; FGF1, fibroblast growth factor 1; FGF7, fibroblast growth factor 7; FOXO1, forkhead box O1; IGF1, insulin-like growth factor 1; IKKβ, inhibitor of nuclear factor kappa B kinase subunit beta; KLF6, KLF transcription factor 6; OSA, obstructive sleep apnea; PDCD4, programmed cell death 4; PTEN, phosphatase and tensin homolog; SATB2, SATB homeobox 2; SIRT1, sirtuin 1; SP1, Sp1 transcription factor; SPRED1, sprouty related EVH1 domain containing 1; TP53, tumor protein P53; VEGF, vascular endothelial growth factor.

The study of Ren et al. (2018) disclosed the upregulation of *miRNA-31* emerging from intermittent hypoxia that resulted in cardiac hypertrophy. As predicted, myocardial remodeling was affected by the *miRNA-31/PKC-ε* signaling pathway to some extent. In turn, in a separate study, the downregulation of *miRNA-31* elicited a protective cardiac performance via *miRNA-31*/PKC-ε/NF-κB pathway (Wang et al., 2015). Despite being engaged in detrimental cardiac changes, the impact of *miRNA-31* dysregulation on hypoxia-subjected tissues seems to be variable. Hypoxic diabetic adipose stem cells (ADSCs) engaged in soft tissue repair processes showed better responses to damage in comparison to non-hypoxic ADSCs (Wang J. et al., 2021). Upregulation of *miRNA-31* in hypoxic ADSCs may account for accelerated wound healing via targeting factor-inhibiting HIF-1 (FIH-1) and epithelial membrane protein-1 (EMP-1) (Huang et al., 2021). Exosomic *miRNA-31* overexpression in recalcitrant diabetic wounds contributed to augmented healing due to its proangiogenic and proliferative features. Likewise, FIH-1 was the estimated target gene (Yan et al., 2022). The study of Han et al. (2021) carried out on healthy skin samples showed a positive impact of overexpressed *miRNA-31* on the cell migration as well. Although the upregulation of *miRNA-31* in diabetic foot skin did not achieve statistical significance due to the variability of samples and the absence of severity-related group division, there is a strong presumption of the influence of *miRNA-31* on skin dysfunction in the course of diabetes mellitus (Ramirez et al., 2015). Given the fact that OSA patients generally have worse progress in diabetic ulcer treatment, the protective effect of upregulated *miRNA-31* on wound healing may work only in some cases or be a part of a specific subset responsible for the repair (Maltese et al., 2018). The same miRNA was upregulated in the retina (Kovacs et al., 2011) and periodontal ligament (Zhen et al., 2017) of diabetic rats, and in endothelial progenitor cells obtained from T2DM patients (Lian et al., 2018). Looking at the last two examples, a plausible common target of *miRNA-31* is a special AT-rich sequence-binding protein 2 (Satb2), which has been reported to trigger vascular endothelial dysfunction and suppression of osteogenic differentiation in DM. Increased levels of transforming growth factor β (TGF-β) occurring in OSA (Hernández-Jiménez et al., 2017) can downregulate Satb2, thereby decreasing bone density via promoting osteoblast dysfunction (Freude et al., 2012). Upregulation of *miRNA-31* and TGF-β demonstrated in OSA coupled with DM may be responsible for the higher incidence of vascular malfunction and poor bone remodeling.

Dysregulation of *miRNA-155*, which has been broadly described in the literature about diabetes and its complications, might be linked with OSA-related kidney deterioration. In renal tissue exposed to chronic intermittent hypoxia, the activation of the NLRP3 inflammasome pathway led to miRNA-155 upregulation. A prompt response to stimuli triggered hypoxia-induced renal injury due to the exacerbation of the inflammatory process (Wu et al., 2018). *miRNA-155* upregulation was detected both in urine and renal tissue, indicating a possible application for this biomarker, albeit the direction of dysregulation may be dependent on the location of miRNA (Akhbari et al., 2019). *miRNA-155* deficiency can favor the acetylation of nephrin, thus ameliorating diabetes-induced perturbation via restoring podocyte function(Lin et al., 2015). Potential corresponding targets in hyperglycemia-injured renal tissue comprise brain-derived neurotrophic factor (BDNF), which has been proven to contribute to kidney deterioration through autophagy attenuation, fibrosis progression, oxidative stress imbalance, and microinflammation (Gao et al., 2022). In several studies dysregulation of BDNF was observed among OSA patients (Arslan et al., 2021; Gabryelska et al., 2022, 2023; Gabryelska and Sochal, 2022). Furthermore, patients suffering from both OSA and T2DM may be more prone to develop impairment of kidney function as a long-term complication; however, this link has not been proven yet and shows the need for further research.

Alteration of *miRNA-126* expression detected in OSA patients (Yang et al., 2018) might be involved in diabetes mellitus development and dysglycemia-related complications as well. In the foreseeable future, *miRNA-126* dysregulation may serve as an accurate diagnostic biomarker of prediabetes (Liu et al., 2014) and T2DM (Dehghani et al., 2020) due to its close correlation. Except the potential ethnic variability (Weale et al., 2021), the progressive decline of miRNA can reflect the advancement of the disease and predict long-term all-cause mortality (Pordzik et al., 2021). Thus, *miRNA-126* downregulation, occurring secondary to OSA, may exacerbate T2DM or even contribute to the onset of the disease. *miRNA-126* downregulation has been reported to impair vascular performance in T2DM (Jansen et al., 2016). It plays an especially important role in the development of retinopathy through the enhancement of neovascularization; targeting vascular endothelial growth factor (VEGF), which in turn promotes migration and sprouting in retinal vascular endothelial cells via VEGF/PI3K/AKT signaling pathway. Additionally, in hypoxia-treated cells, *miRNA-126* is a negative regulator of VEGF expression (Ye et al., 2013). It may partly explain why OSA subjects exhibit repercussions linked with eyes, such as increased retinal vessel tortuosity, declined vascular density, or pathological changes in the choroid (Nakayama et al., 2021). *miRNA-126* downregulation is likewise associated with diabetic nephropathy (Al-Kafaji et al., 2016) and worse diabetic foot ulcer healing (Zhang J. et al., 2017). As diabetic patients with OSA are generally more vulnerable to developing diabetic retinopathy (Chang et al., 2018) and nephropathy (Misra and Shrivastava, 2016) or have impaired wound healing (Ramirez et al., 2015), these dependencies are of great importance.

*miRNA-181a* is one of the most hypoxia-sensitive miRNAs which is best illustrated by the example of tumor progression (Agrawal et al., 2014; Sun et al., 2015a; Macharia et al., 2021). Under these circumstances, the main mechanism of action is presumably aimed at angiogenesis promotion via targeting VEGF, which then induces tumor growth and subsequent metastasis development (Sun et al., 2015b; Silvestris et al., 2017). Dysregulation of *miRNA-181a* in response to hypoxia may participate in cardiovascular damage where the possible interplay between *miRNA-181a*, VEGF, and HIF1α seems to be important (Cuevas et al., 2014). The underlying role of *miRNA-181a* in cell damage and apoptosis via SIRT1 regulation can also be observed in injured cardiomyocytes (Qi et al., 2020; Song et al., 2021). *miRNA-181a* also plays a role in the development of cerebral ischemia, having a neuroprotective effect when upregulated. What is more, in OSA patients the expression of *miRNA-181a* is decreased and correlates with the AHI and arousal index. Among diabetic patients, the expression of *miRNA-181a* in the adipose tissue is reduced as well and a higher level of this is known to prevent insulin resistance induced via TNFα (Lozano-Bartolomé et al., 2018). The exact impact of *miRNA-181a* on diabetes mellitus development is not fully explored, presenting different data. In Gok et al. (2019) study the downregulation of *miRNA-181a* and simultaneous upregulation of SIRT1 were connected with the development of T2DM. However, Zhou et al. (2012) showed that the downregulation of *miRNA-181a* improves hepatic sensitivity via SIRT1 upregulation; SIRT1, as a gene with a positive therapeutic effect on glucose metabolism (Sun et al., 2007), may play a compensative role during diabetes mellitus development. Decreased levels of *miRNA-181a* were also noticed in diabetic complications including cardiomyopathy (Raut et al., 2016; Zhao S. F. et al., 2021) and nephropathy (Zha et al., 2019; Liang and Xu, 2020). As the case may be, in diabetic hearts *miRNA-181* targets programmed cell death 4 (PDCD4), or TP53. Cardiomyocytes with decreased *miRNA-181a* levels are more prone to develop hypertrophy, inflammation, impaired angiogenesis, and undergo apoptosis. In diabetic kidney tissue, *miRNA-181a* targets may be a Kruppel-like factor 6 (KLF6) and early growth response factor-1 (EGR-1), which are involved in the disordered proliferation of the glomerular mesangial cells, tubulointerstitial fibrosis, and enhanced cell apoptosis. Overall, the downregulation of *miRNA-181a* observed in OSA may mean that non-OSA patients are more resistant to developing diabetic-related cardiomyopathy or nephropathy, but have less expanded compensatory mechanisms against impaired insulin sensitivity. In regard to other diabetic complications, it was discovered that the upregulation of *miRNA-181a* may be responsible for diabetic corneal nerve neuropathy in mice (Hu et al., 2020). Upregulated levels of *miRNA-181a* were also noticed in patients with gestational diabetes mellitus (Hromadnikova et al., 2020, 2022) and animal diabetic models with impaired wound and fracture healing (Takahara et al., 2018; He et al., 2019).

Another promising hypoxia-regulated molecule is *miRNA-199a*, its activity mainly focuses on the co-expression of two target genes, HIF-1α and SIRT1. HIF-1α compensation permits the cells to adapt to low oxygen conditions. In a hypoxic preconditioning *miRNA-199a* downregulation was associated with the upregulation of HIF-1α and SIRT1, resulting in the adaptation to external stimulus (Rane et al., 2009). A similar outcome was obtained after stimulation of the insulin receptor, which regulates the AKT pathway (Rane et al., 2010). SIRT1, besides maintaining metabolic homeostasis, acts as a regulator of HIF-1α. SIRT1 binds to the protein and deacetylates lysine 674, resulting in the suppression of HIF-1α transcriptional activity (Lim et al., 2010). Some authors describe SIRT1 as an indispensable element of HIF-1α activity due to its role in the positive regulation of the protein (Laemmle et al., 2012). In OSA patients Santamaria-Martos et al. (2019b) revealed downregulation of *miRNA-199a*. It was also seen that induction of *miRNA-199a* and ensuing HIF-1α downregulation can relieve OSA-related hypertension via oxidative stress injury reduction and suppression of inflammation (Guo et al., 2022). In diabetic subjects, a downregulation of *miRNA-199a* was observed in nephropathy and the inhibitor of nuclear factor kappa b kinase subunit beta (IKKβ) was identified as a potential target (Zhang R. et al., 2020). Additionally, IKKβ can play an important role in developing insulin resistance (Zhao L. et al., 2021) and atherosclerosis (Imamura et al., 2016) in OSA patients. Decreased levels of *miRNA-199a* were similarly present in diabetic-induced cardiomyopathy via targeting protein kinase B (AKT) and growth factors: vascular (VEGF), insulin-like (IGF), and acidic fibroblast (FGF-1) (Ahmed et al., 2021). Diabetic cataract was associated with *miRNA-199a* downregulation and its influence on the specific protein 1 (SP1) gene (Liu et al., 2020). In turn, diabetic retinopathy can partially result from *miRNA-199a* dysregulation associated with VEGF or FGF7 signaling (Wang L. et al., 2020; Zhou et al., 2021). In contrast, Yan et al. (2014) found out that *miRNA-199a* levels were upregulated in T2DM patients and due to the influence on GLUT4 expression might be involved in insulin resistance development. Overexpression of TNF-α presented in OSA may additionally enhance DM development by suppressing GLUT-4 expression and promoting insulin resistance through TNF-α/IKKβ/IKβ/NF-κB signaling pathway (Swaroop et al., 2012; Ji et al., 2021). Upregulation of *miRNA-199a* might be connected with decreased cell viability and enhanced apoptosis in pancreatic beta cells through the downregulation of SIRT1 (Lin et al., 2017). In accordance with other articles, a high level of *miRNA-199a* was observed in the placenta of gestational diabetes mellitus patients(Guan et al., 2022) and tissues obtained from patients with a diabetic foot ulcer (Wang H. et al., 2022). *miRNA-199a* expression in patients with diabetic neuropathy showed inconsistent results; the downregulation was associated with increased binding immunoglobulin protein expression levels (Hassani et al., 2023), while upregulation decreased the levels of SerpinE2 (Li et al., 2017).

**Figure 2:**
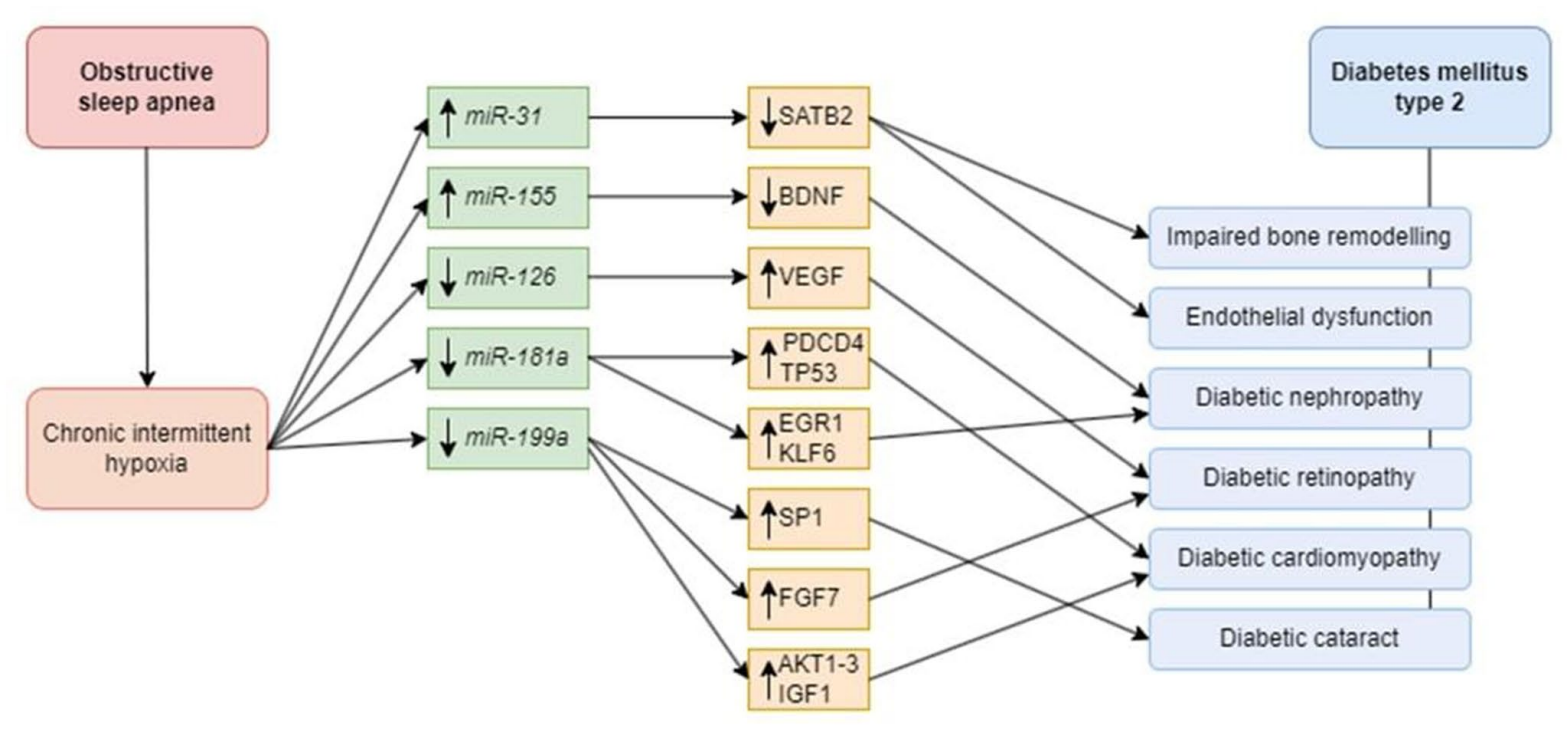
Possible role of microRNAs in the pathophysiology of diabetes mellitus and diabetic complications. Chronic intermittent hypoxia as the main consequence of obstructive sleep apnea affects *miR-31* and -*155*, resulting in their downregulation, and *miR-126, -181a*, and -*199a*, leading to their upregulation. Dysregulation of microRNAs can predispose patients suffering both from OSA and DM to particular complications. Decreased level of SATB2 contributes to impaired bone remodeling and endothelial dysfunction. In turn, BDNF dysregulation is associated with diabetic nephropathy. Downregulation of the VEGF gene results in diabetic retinopathy development. Increased TP53 and PDCD4 lead to diabetic cardiomyopathy. Targeting EGR-1 and KLF6 can trigger diabetic nephropathy. An increase in SP1 is responsible for diabetic cataract. Another upregulated target, FGF7, can contribute to diabetic retinopathy development. Downregulation of AKT, IGF, and FGF-1 play important role in diabetic cardiomyopathy development. miR, microRNA; SATB2, special AT-rich sequence-binding protein 2; BDNF, brain-derived neurotrophic factor; VEGF, vascular endothelial growth factor; PDCD4, programmed cell death 4; TP53, tumor protein 53; EGR1, early growth response 1; KLF6, Kruppel-like factor 6; SP1, specific protein 1; FGF7, fibroblast growth factor 7; AKT, protein kinase B; IGF, insulin-like growth factor 1; FGF1, fibroblast growth factor 1.

Detection of described miRNAs with an altered expression profile in OSA patients may provide valuable info about a high risk of T2DM development as an OSA complication in such patients (Figure 2).

## 3. Conclusion

OSA is associated with metabolic complications such as metabolic syndrome and T2DM. Many of the possible molecular pathways involved in the relationship between OSA and metabolic complications have been described in the literature. In this review, we summarized the available data about the role of miRNAs in OSA-related metabolic disorders development. The literature suggests that OSA alters the expression of miRNAs in the organism. It results in altered gene expression. The most important examples include *miRNA-181a* and *miRNA-199a*, which play an important role in the metabolic consequences of OSA development. Future miRNA investigations in the context of hypoxia should focus on the above-mentioned miRNAs. They can act not only as cheaper and more reliable OSA diagnostic markers but also valuable prognostic factors in patients suffering from OSA. For a better understanding of the relationship between OSA and metabolic complications, studies should also focus on the possible change in miRNA levels in response to implemented treatment of T2DM and MetS, and their mechanisms of action.

## Author contributions

FFK and AG provided the overall concept and framework of the manuscript. JJ, MM, and FFK researched and identified appropriate articles and wrote the manuscript. JJ and MM were responsible for the visualization. AG, BS, PB, DS, and MS revised the manuscript. All authors have read and agreed to the published version of the manuscript.

## Funding

The study was supported by National Science Centre, Poland, Preludium 20 grant no. 2021/41/N/NZ5/00486 (for FFK).

## Conflict of interest

The authors declare that the research was conducted in the absence of any commercial or financial relationships that could be construed as a potential conflict of interest.

## Publisher’s note

All claims expressed in this article are solely those of the authors and do not necessarily represent those of their affiliated organizations, or those of the publisher, the editors and the reviewers. Any product that may be evaluated in this article, or claim that may be made by its manufacturer, is not guaranteed or endorsed by the publisher.
